# The Toxic Effects of Urotropin

**Published:** 1904-09-10

**Authors:** 


### THE TOXIC EFFECTS OF UROTROPIN.

Experience has fully shown, as is the case with most new pharmacological preparations, that urotropin cannot be administered to patients in comparatively large doses with that freedom from anxiety which was at first thought possible. As time goes by fresh examples are repeatedly coming to hand of cases where urotropin has given rise to toxic effects which in some instances have been sufficient to cause alarm. It is important that this fact should be borne in mind, for, in using the drug as a urinary antiseptic, there is sometimes a tendency to give unduly large doses in order to get a high percentage in the urine. Two cases have recently come under our observation, in which hæmaturia followed 10-grain doses of urotropin, and in one of these instances the hæmorrhage was profuse. Dr. W. Coleman,[1] as the result of an extended search into the literature of the subject, mentions the following poisonous manifestations due to the drug. (1) Irritation of the stomach as shown by impairment of digestion and slight malnutrition. (2) Diarrhœa and slight abdominal pain. (3) Irritation of skin, with a rash resembling that of measles. (4) Headache and tinnitus aurium. (5) Albuminuria. (6) Irritation of the bladder, shown by strangury. (7) Hæmaturia and hæmoglobinuria. To lessen the chance of producing poisonous symptoms the drug should be freely diluted, five grains being given in half a pint of water; and if fixed rules are in any case desirable in therapeutics this is a harmless and useful one to follow, for it insures against producing an excessive percentage of urotropin in the patient's urine. Personally we have never ordered a patient more than ten-grain doses and we have had reason to be satisfied with the results, although M. Lubowski[2] characterises such a quantity as "remarkably small" and as the "lowest effective dose."

[1] Med. News, 1903, p. 394. [2] Therapist, Aug. 15.

